# Habitual Green Kiwifruit Consumption Is Associated with a Reduction in Upper Gastrointestinal Symptoms: A Systematic Scoping Review

**DOI:** 10.1093/advances/nmac025

**Published:** 2022-03-10

**Authors:** Simone B Bayer, Chris M Frampton, Richard B Gearry, Giovanni Barbara

**Affiliations:** Gastrointestinal Unit for Translational Studies, Department of Medicine, University of Otago Christchurch, Christchurch, New Zealand; Biostatistics and Computational Biology Unit, University of Otago Christchurch, Christchurch, New Zealand; Gastrointestinal Unit for Translational Studies, Department of Medicine, University of Otago Christchurch, Christchurch, New Zealand; Department of Medical and Surgical Sciences, University of Bologna, Bologna, Italy; IRCCS Azienda Ospedaliero-Universitaria, Bologna,Italy

**Keywords:** reflux, dyspepsia, indigestion, abdominal pain, bloating

## Abstract

Kiwifruit have known positive effects on digestion. During clinical intervention trials using kiwifruit to improve constipation, upper gastrointestinal (GI) symptoms such as abdominal discomfort and pain, indigestion, and reflux were also alleviated. We aimed to evaluate the evidence for upper GI symptom relief by kiwifruit in clinical trials on participants with functional constipation (FC), irritable bowel syndrome with constipation (IBS-C), and healthy participants, and to elucidate which symptoms may be relieved and whether a difference exists between the effects of gold and green kiwifruit. We executed a systematic scoping review of 3 electronic databases from 1947 through January 2021 to identify clinical trials that reported effects of green or gold kiwifruit or kiwifruit compounds on upper GI symptoms as secondary outcomes in healthy participants or participants with FC or IBS-C. Studies were divided into those using the Gastrointestinal Symptom Rating Scale (GSRS) and those using alternative measurement tools. GSRS outcomes were pooled and statistically analyzed; non-GSRS outcomes were summarized. We identified 12 clinical trials with a total of 661 participants (124 controls, 537 receiving intervention) providing evidence for symptom relief of upper GI symptoms by kiwifruit intake. Only 5 of the 12 clinical trials used the GSRS to assess upper GI symptom relief. We found good evidence that green kiwifruit may reduce abdominal discomfort and pain, and some evidence that kiwifruit consumption may attenuate indigestion. Pooled GSRS outcome analysis indicates an average reduction of –0.85 (95% CI: –1.1, –0.57; *Z* = 6.1) in abdominal pain scores and –0.33 (95% CI: –0.52, –0.15; *Z* = –3.5) in indigestion scores with habitual kiwifruit consumption. While the number of studies reporting on upper GI symptom relief with a comparable measurement is limited, there is consistent evidence for the efficacy of kiwifruit on upper GI symptom relief. More research to strengthen the evidence is recommended.

## Teaser

**Statement of Significance**: This is the first review showing evidence that habitual consumption of kiwifruit may improve upper gastrointestinal symptoms such as abdominal pain and dyspepsia.

## Introduction

### Rationale

For many years, the green kiwifruit *Actinidia deliciosa* (cultivar Hayward) and the gold kiwifruit *Actinidia chinensis* (cultivar Zesy002) have been extensively studied for their positive effects on physical and mental health (1–3). Both cultivars are nutrient-rich and contain high amounts of vitamin C, dietary fiber, folate, potassium, and antioxidants (4–6). In addition, both cultivars possess the proteolytic enzyme actinidin (7) at similar concentrations, albeit the activity differs (8). The proteolytic activity of green kiwifruit actinidin is 8 times higher than that of Zesy002 (8). The now-extinct gold kiwifruit cultivar Hort16 does not possess actinidin activity. Actinidin may provide antimicrobial protection to the plant (9) and is able to enhance digestion of beef, dairy, and wheat (10–12). Actinidin is also implicated to accelerate gastric emptying (13, 14) and it is theorized it may modulate pain reception and anti-inflammatory responses of the gut through protease-activated receptors (15).

The major focus of clinical studies has been the positive effects of kiwifruit on digestive health and comfort, specifically on symptoms of constipation (15). Kiwifruit increase bowel movement frequency (3, 16–20), reduce straining (16–18, 21), improve stool consistency (16–18, 22–25) without negative side effects (16, 18, 26), and are preferred by study participants with constipation (25).

Constipation refers to the definition of 2 common, chronic, separate, albeit frequently overlapping, functional gastrointestinal (GI) disorders (FGIDs)—namely, functional constipation (FC) and irritable bowel syndrome with constipation (IBS-C) (27). The main difference between FC and IBS-C is the presence of abdominal pain (27) in the latter. Although the pathogenesis of pain is poorly understood, it may involve visceral hypersensitivity to normal physiological stimuli, such as distension (28). Another common symptom of FC as well as IBS-C is bloating (27, 29).

Both IBS-C and FC belong to the bowel cluster of FGIDs (27). FGIDs can affect any part of the GI tract, but have no significant structural, mucosal, or inflammatory component (27, 30–34). They include disorders like functional chest pain, functional heartburn, reflux hypersensitivity, functional dyspepsia, functional dysphagia, functional diarrhea and diarrhea-predominant irritable bowel syndrome (IBS), belching disorders, as well as FC and IBS-C, among others (27, 30, 32–34). FGIDs are common; in a global study on FGID prevalence from 2020, up to 40.7% of the population could be diagnosed with an FGID (35). FGIDs are more common in women, and can arise at any age (35). They are associated with psychological comorbidity, encompassing mainly anxiety and depression (34, 36–38), lower quality of life (39, 40), and increased health care utilization and seeking for both primary and specialist care (35, 41).

There is wide overlap between FC and IBS-C (42). In addition, bowel disorders may overlap with functional gastroduodenal disorders (27, 34, 35). This overlap includes pathophysiological mechanisms such as visceral hypersensitivity to physiological stimuli (33) as well as symptom patterns including bloating (33, 43) and belching (33). This overlap also means that many patients can be diagnosed with more than 1 FGID (43, 44). The most well-described association is between functional dyspepsia and IBS (43, 45).

Interestingly, kiwifruit were described as a treatment for dyspepsia in an early Chinese pharmacopeia from the Tang Dynasty (6). Furthermore, in some clinical trials using kiwifruit for constipation management, positive effects on upper GI symptoms such as dyspepsia were regularly reported (16–18, 23).

However, it is uncertain which upper GI symptoms may be relieved by kiwifruit intake, if there is a difference in the observed effects between green and gold kiwifruit, and if the quality of the existing evidence is adequate to support a recommendation for kiwifruit to help manage upper GI symptoms.

### Objectives

The Preferred Reporting Items for Systematic Reviews and Meta-Analyses (PRISMA) guidelines characterize the synthesis of evidence and assessment of the scope on a topic as a scoping review. Therefore, we conducted a systematic scoping review to gain an overview of all studies that used whole kiwifruit, freeze-dried kiwifruit supplements, and kiwifruit extracts as interventions in relation to digestion or digestive health and reported upper GI symptom relief as secondary outcomes in healthy participants or participants with FC or IBS-C, and to determine the extent and the quality of evidence supporting effects on upper GI symptoms. We aimed to answer the question, What evidence is already available concerning the improvement in upper GI symptoms following consumption of green or gold kiwifruit?

## Methods

### Protocol and registration

This systematic scoping review was performed adhering to the Preferred Reporting Items for Systematic Reviews and Meta-Analyses for Scoping Reviews (PRISMA-ScR) guidelines. A protocol for the systematic scoping review has been registered with the Center for Open Science (doi: 10.17605/OSF.IO/CQZNA).

### Selection criteria

For the systematic scoping review, we included clinical trials on GI effects of green or gold kiwifruit or kiwifruit compounds that also reported on upper GI symptoms as secondary outcomes in healthy participants or participants with FC or IBS-C. Published peer-reviewed journal manuscripts and unpublished reports were included if they were written in English, German, or Spanish. We excluded reviews, papers reporting on other types of kiwifruit, uncharacterized extracts or fruit mixtures, and studies covering allergic-type reactions.

### Information sources

We searched PubMed to 21 January, 2021, Ovid Embase® from 1947 to 21 January, 2021, and Ovid MEDLINE® (including Epub Ahead of Print, In-Process & Other Non-Indexed Citations, Daily and Versions) from 1946 to 22 January, 2021 for identification of relevant studies. Supplementary studies were determined by scanning relevant reviews, and unpublished reports were identified through discussions with key individuals and provided by the funder, Zespri® International Ltd.

### Search and selection of sources

Search items included “Kiwifruit” AND “Digestion” OR “Intestinal” OR “Intestine” OR “GI” OR “Gastrointestinal tract” OR “Gut” OR “Bowel” OR “Functional colonic disease” OR “Constipation” OR “Irritable bowel syndrome” OR “IBS” OR “Irritable colon.” The search was conducted by the primary author, who also reviewed the titles and abstracts of all journal papers to exclude manuscripts outside of the stated selection criteria. Full texts of the remaining studies were assessed to establish whether inclusion criteria were met and whether the relevant information in the studies was suitable for the systematic scoping review. When relevant conference abstracts were identified, the corresponding author and/or the funder (Zespri® International Ltd.) were contacted with a request for missing data.

All 85 of the identified full-text studies were screened and evaluated by the primary author for this review.

### Data charting and information assessment

A data charting form was developed by the primary author. The variables encompassed author, year, country, title, design/type of study, interventions used, intervention duration, aims, number of participants in each group and gender distribution, the methods used, definition used for IBS and FC determination, details of primary and secondary outcomes, and, when relevant, conclusion.

### Synthesis of results

Studies reporting on upper GI secondary outcomes were divided into those using the Gastrointestinal Symptom Rating Scale (GSRS) and those that used alternative measurement tools. We summarized the upper GI non-GSRS outcomes along with the method used to determine the outcome. GSRS outcomes that are reported on in this review are total GRSR score and the relevant domains abdominal pain, indigestion, and reflux.

### Statistical analysis

The estimation for the changes in the GSRS domains and totals were extracted from the published and unpublished reports. Pooled estimates were derived from these aggregate summaries using a random-effects model for each of the interventions and control groups. Those estimates were compared using a *Z* test and 95% CIs calculated for the differences using Microsoft Excel® (Microsoft Corporation) and IBM SPSS® Statistics version 25 (IBM Corporation) and were used for analysis.

## Results

### Selection of sources of evidence

**FIGURE 1:**
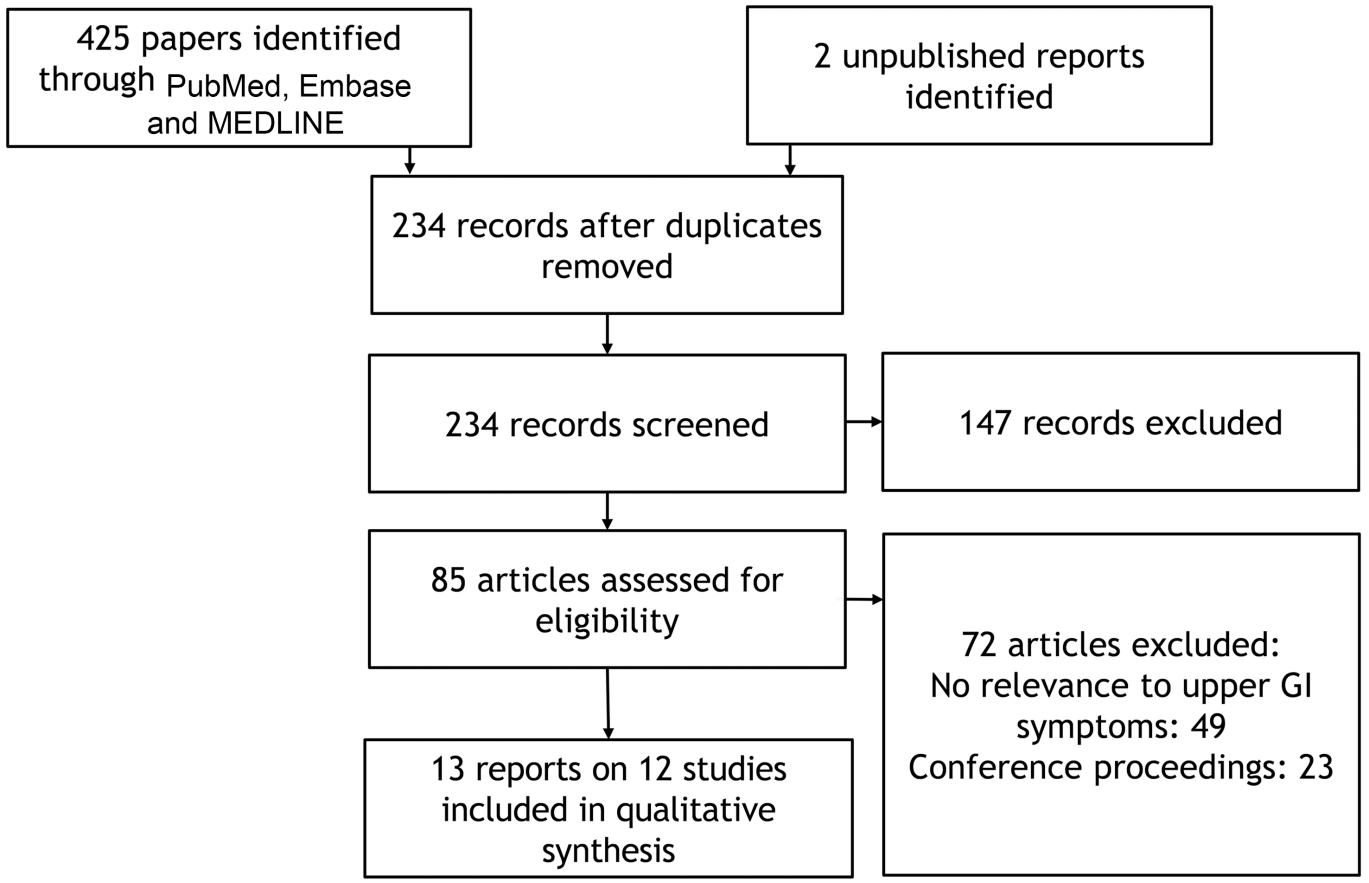
Study selection flowchart. GI, gastrointestinal.

We identified 234 relevant records on kiwifruit, and assessed 85 articles for eligibility and relevance. As a result, we included 13 manuscripts on 12 studies (*n* = 661 participants; 154 controls and 507 with constipation symptoms) reporting on upper GI effects of kiwifruit (16–18, 21–26, 46–53). A flowchart showing study selection is shown in **Figure 1**.

### Characteristics and quality of sources

The baseline characteristics of the used studies for the scoping review section are presented in **Table 1**. Four studies used powdered kiwifruit supplements as the intervention, 5 used the green kiwifruit cultivar “Hayward,” and 3 studies used gold kiwifruit. The intervention dosage ranged from 2 kiwifruit/d in 5 studies to 3 kiwifruit/d in 3 studies. The intervention time ranged from 1 to 28 d. Of the studies, 3 were parallel design, 1 was an open-label design, and 8 were crossover designs. Seven trials were conducted in New Zealand, 2 in Spain, 2 in the United States, and 1 study in Italy, Japan, and China, respectively.

**TABLE 1:** Description of clinical trials included in the systematic scoping review reporting upper gastrointestinal symptom relief^1^

			Duration of intervention	Number of subjects					
First author (ref)	Year	Intervention details		Controls (M/F)	Cases (M/F)	Location	Primary outcome	GSRS	Rome criteria
Kiwifruit supplements									
Weir (26)	2018	Zyactinase™ (Vital Food Processors Ltd.) 2160 mg/d vs. placebo (isomalt)	7 d	*n* = 0	FC, *n* = 58 (NA)	China	Bowel movement frequency	No	Rome III
Kindleysides (46)	2015	Kivia powder (Vital Food Processors Ltd.) 2 × 1000 mg/d vs. placebo	3 wk	*n* = 0	FC, *n* = 32 (3/29)	New Zealand	Bowel movement frequency	Yes	No
Ansell (47), Eady (48)	2014	Actazin (Anagenix Ltd.) 600 mg/d, Actazin 2400 mg/d, Gold (Anagenix Ltd.) 2400 mg/d vs. placebo	4 wk	*n* = 19 (2/17)	FC, *n* = 9 (1/8)	New Zealand	Bowel movement frequency	No	Rome III
Udani (24)	2013	Kivia powder (Vital Food Processors Ltd.), 1 sachet (Zyactinase™ 5.5 g)/d, vs. Spirulina	4 wk	*n* = 0	FC, *n* = 87 (32/55)	USA	Bowel movement frequency	No	Rome III
Green kiwifruit									
Caballero (22)	2020	2 kiwifruit + low-flatulogenic diet vs. low-flatulogenic diet only	2 wk	*n* = 11 (3/8)	*n* = 0	Spain	Intestinal gas transit	No	No
Wallace (49)	2017	200 g kiwifruit vs. 200 g Hort16A kiwifruit after ingestion of 250 g beef meat	1 d	*n* = 10 (10/0)	*n* = 0	New Zealand	Changes in gastric-emptying rate, gastric pH, temperature, and small bowel transit times	No	No
Drummond (16)	2020	2 green kiwifruit/d vs. 7.5 g psyllium/d	4 wk	*n* = 63 (26/37)	FC+IBS-C, *n* = 121 (22/99)	New Zealand, Italy, Japan	Bowel movement frequency	Yes	Rome III
Chey (25)	2020	2 green kiwifruit/d vs. 100 g prunes/d vs. 12 g psyllium/d	4 wk	*n* = 0	FC, *n* = 79 (10/69)	USA	Bowel movement frequency	No	No
Cunillera (21)	2015	3 kiwifruit/d	3 wk	*n* = 0	FC, *n* = 46 (4/42)	Spain	Bowel movement frequency	No	Rome III
Gold kiwifruit									
Eady (23)	2020	3 kiwifruit/d, with vs. without skin	4 wk	*n* = 19	IBS-C, *n* = 19 (11/27)	New Zealand	Changes in inflammatory cytokines	Yes	Rome III
Eady (17)	2019	3 kiwifruit/d vs. 2.5 teaspoons psyllium/d	4 wk	*n* = 0	FC+IBS-C, *n* = 32 (0/32)	New Zealand	Bowel movement frequency	Yes	Rome III
Bayer (18)	2021	2 kiwifruit/d vs. 7.5 g psyllium/d	4 wk	*n* = 32 (6/26)	FC+IBS-C, *n* = 24 (2/22)	New Zealand	Bowel movement frequency	Yes	Rome IV

1 FC, functional constipation; GSRS, Gastrointestinal Symptoms Rating Scale; IBS-C, irritable bowel syndrome with constipation; ref, reference.

Of 661 participants, a total of 537 received interventions: 131 received supplement capsules and 406 received kiwifruit, of whom 126 participants received gold kiwifruit and 280 received green kiwifruit. The total number of participants per study ranged from 10 to 184, with 472 participants being female, which accounts for 77.9% of participants; 1 study did not report on participant gender (26). Two studies were conducted in healthy participants only; 6 studies had no control group. In 1 study only individuals with IBS-C participated, in 3 studies the participants had IBS-C and FC, and in 6 studies the participants were characterized as FC. Four trials did not use the Rome criteria to characterize participants, 7 trials used Rome III criteria, and 1 trial used Rome IV criteria.

An increase in bowel movement frequency was the most common primary outcome (9 studies); in 1 study the primary outcome was changes in inflammatory cytokines, in 1 study the primary outcomes included increased gastric-emptying rate and reduced small bowel transit time, and in 1 study the primary outcome was intestinal gas transit. Seven studies used nonvalidated tools or additional questions to the bowel movement survey to determine additional GI outcomes, and 4 used validated tools (see **Table 2**). The validated tools included the GSRS (*n* = 5) (54), the Birmingham IBS Survey (*n* = 2) (55), the Visual Analog Scale for IBS (VAS-IBS; *n* = 1) (56), the Patient Reported Outcome Information System GI symptom domains (*n* = 1) (57), and the Structured Assessment of GastroIntestinal Symptoms (SAGIS; *n* = 1) (58).

**TABLE 2:** Reported outcomes with kiwifruit interventions on upper gastrointestinal symptoms other than GSRS^1^

First author (ref)	Method	Reported outcome
Weir (26)	Abdominal discomfort score based on Rome III (4-point Likert-like scale rating based on intensity of tenesmus, abdominal discomfort, bloating, flatulence, and abdominal pain)	With kiwifruit product, abdominal discomfort scale was significantly reduced during the dosing phase (*P* ≤ 0.01) to near normal, retained during follow-up phase of 7 d
Ansell (47), Eady (48)	Questions about bloating, flatulence, and abdominal pain in addition to bowel frequency and laxative use; Birmingham IBS survey (in unpublished report only)	In the healthy cohort, abdominal pain was not different from baseline (0.04 Actazin low vs. –0.01 Actazin high vs. 0 Gold).In the case cohort, abdominal pain was also not significantly altered (–0.01 Actazin low vs. –0.02 Actazin high vs. –0.06 Gold).In the Actazin high group, participants reported increased flatulence as adverse effects. With the Birmingham IBS survey, abdominal pain was significantly reduced in the case cohort (*P* ≤ 0.004)
Udani (24)	Questions about abdominal bloating, abdominal discomfort or pain, flatulence, satisfaction with bowel movements, and burping in addition to bowel frequency	With kiwifruit product, abdominal discomfort or pain was significantly reduced at week 4 (*P* ≤ 0.018) in comparison to placebo. After 2 wk, reduction in abdominal bloating reached significance (no image or table presented, *P* ≤ 0.003). Flatulence was significantly lower with kiwifruit product than with placebo at weeks 2 and 3 (*P* ≤ 0.047 and *P* ≤ 0.023, respectively)
Caballero (22)	Measurement of rectal gas evacuation, abdominal perception, abdominal distension; questions about abdominal bloating, abdominal discomfort or pain, flatulence, satisfaction with bowel movements, and burping in addition to bowel frequency	Kiwifruit was well tolerated and induced no abdominal pain. There was no difference in gas evacuation or abdominal distension between the groups
Wallace (49)	Gastric and small bowel transit time assessed by SmartPill™Changes in subjective feelings of satiety, assessed by VASChanges in subjective feelings of gastric comfort, assessed by questionnaire	There was no difference in gastric or small bowel transit time between the interventions. There were no differences in subjective feelings of satiety. Bloating was significantly reduced with green kiwifruit (*P* ≤ 0.005 and *P* ≤ 0.028)
Chey (25)	Treatment satisfaction (yes/no) and adverse events assessment	Adverse events were “least common with kiwifruit”: abdominal pain was not reported with kiwifruit (*P* ≤ 0.05). Lowest dissatisfaction score with kiwifruit (*P* ≤ 0.02)
Cunillera (21)	Questionnaire which included some questions about satisfaction with bowel habits	Significant reduction in bloating and swelling (*P* ≤ 0.001). Changes in stomachache did not reach threshold (*P* ≥ 0.052)
Eady (23)	Birmingham IBS Questionnaire	In IBS, kiwifruit with skin reduced abdominal pain significantly (*P* ≤ 0.0055)
Bayer (18)	PROMIS, SAGIS	In all groups and with both interventions, PROMIS disrupted swallowing scores improved significantly (*P* ≤ 0.036), as well as SAGIS epigastric symptom scores (*P* ≤ 0.003) and SAGIS acid regurgitation and gas scores (*P* ≤ 0.001). There was no difference between the interventions reported

1 GSRS, Gastrointestinal Symptoms Rating Scale; IBS, irritable bowel syndrome; PROMIS, Patient-Reported Outcome Measurement Information System; ref, reference; SAGIS, Structured Assessment of Gastrointestinal Symptoms; VAS, visual analog scale.

### Abdominal discomfort and pain

Seven clinical trials reported a significant reduction in abdominal discomfort or pain posttreatment with kiwifruit and kiwifruit supplements as interventions, of which 6 trials had no healthy participants as controls. Three clinical trials omitted the use of control substances; 4 trials used psyllium as a positive control, of which 1 added prunes as an additional control. Five trials opted for negative controls (placebo), of which 1 used the gold kiwifruit variety “Hort16,” which does not contain actinidin. Two trials used kiwifruit supplements and reported a significant reduction in abdominal discomfort in comparison to placebo [*P* ≤ 0.01 (26) and *P* ≤ 0.018 (24)], and 1 study comparing green kiwifruit with prunes and psyllium noted the lowest dissatisfaction score with kiwifruit (25) (*P* ≤ 0.02). The same study also showed a significant lack of abdominal pain as an adverse event (*P* ≤ 0.05). All 3 studies focused on participants with FC only and had no healthy control group (see Table 2 and **Table 3** for details on scores and Table 1 for information on participants). One placebo-controlled clinical trial conducted with a supplement reported both the absence of abdominal pain in case and healthy control groups, as well as a significant reduction in abdominal pain scores with the Birmingham IBS survey (48) (*P* ≤ 0.004) posttreatment; however, the case group comprised only 9 participants with FC, and there was no statistical difference between the interventions. Another study also reported the absence of abdominal pain in healthy controls consuming 2 green kiwifruit/d alongside a low-flatulogenic diet (22). This trial was compared with the low-flatulogenic diet only with no placebo for the kiwifruit. In a Spanish trial (21) in participants with FC (*n* = 46; no healthy controls, no positive or negative control), which used 3 green kiwifruit/d, a reduction in abdominal pain was observed but did not reach significance (*P* ≥ 0.052).

**TABLE 3:** Overview of existing GSRS mean Likert-scores with kiwifruit interventions^1^

			Total GSRS^2^		Reflux		Abdominal pain		Indigestion	
First author (ref)	Group (*n*)	Results reported as	Mean [95% CI]	*P* or *Z* value	Mean [95% CI]	*P*	Mean [95% CI]	*P*	Mean [95% CI]	*P*
Kindleysides (46)^3^	FC (32)	Difference^4^	–0.3	0.0204	—	—	—	—	—	—
Drummond (16)	Controls (63)	Difference	–0.08 [–0.20, 0.05]	0.2186	–0.05 [–0.22, 0.13]	0.5872	–0.02 [–0.18, 0.14]	0.7738	–0.16 [–0.30, –0.02]	0.0277
	FC (60)	Difference	–0.22 [–0.32, –0.12]	≤0.0001	–0.17 [–0.34, 0.00]	0.0509	–0.07 [–0.29, 0.15]	0.5406	–0.22 [–0.39, –0.04]	0.0173
	IBS-C (61)	Difference	–0.36 [–0.52, –0.21]	≤0.0001	–0.18 [–0.32, –0.04]	0.0127	–0.30 [–0.55, –0.04]	0.0237	–0.40 [–0.64, –0.16]	0.0016
	FC+IBS-C (121)	Difference	–0.30 [–0.40, –0.20]	≤0.0001	–0.18 [–0.29, –0.07]	0.0015	–0.19 [–0.36, –0.02]	0.0267	–0.31 [–0.46, –0.16]	≤0.0001
Eady (23)	Controls (20)	Baseline	1.228	—	1.13 ± 0.23	—	1.25 ± 0.24	—	1.37 ± 0.34	—
		Kiwifruit flesh	1.170	—	1.13 ± 0.23	—	1.11 ± 0.19	—	1.36 ± 0.41	—
		Difference^4^	–0.058 ± 0.28	—	0.00 ± 0.23	—	–0.14 ± 0.22	—	–0.01 ± 0.38	—
		Kiwifruit flesh + skin	1.226	—	1.16 ± 0.34	—	1.28 ± 0.39	—	1.43 ± 0.49	—
		Difference^3,4^	–0.002	—	0.03	—	0.03	—	0.06	—
	IBS-C (20)	Baseline	2.138	—	1.55 ± 0.91	—	2.05 ± 0.89	—	2.48 ± 1.11	—
		Kiwifruit flesh	1.786	—	1.34 ± 0.57	—	1.67 ± 0.95	—	2.10 ± 1.26	—
		Difference^4^	–0.35 ± 1.07	—	–0.21 ± 0.76	—	–0.38 ± 0.92	0.036 c.t.t	–0.38 ± 1.19	—
		Kiwifruit flesh + skin	2.042	—	1.56 ± 0.91	—	2.02 ± 1.12	—	2.44 ± 1.28	—
		Difference^3,4^	–0.096	—	0.01	—	–0.03	—	–0.04	—
Eady (17)	FC+IBS-C (32)	Difference	–0.378 ± 0.97	—	–0.08 ± 0.67	—	–0.31 ± 0.66	≤0.05	–0.67 ± 0.88	0.002
Bayer (18)	Controls (32)	Difference	0.028	—	–0.05 [–0.27, 0.17]	—	0.03 [–0.28, 0.34]	—	–0.02 [ –0.25, 0.20]	—
	FC (11)	Difference	–0.402	—	–0.08 [–0.43, 0.27]	—	–0.31 [–0.80, 0.19]	—	–0.54 [–0.90, –0.18]	0.008 c.t.b
	IBS-C (13)	Difference	–0.106	—	–0.41 [–0.77, –0.04]	—	–0.09 [–0.61, 0.43]	—	–0.27 [–0.65, 0.10]	0.008 c.t.b
	FC+IBS-C (24)	Difference	–0.272	—	–0.24 [–0.49, 0.01]	—	–0.20 [–0.56, 0.15]	—	–0.41 [–0.67, –0.15]	0.008 c.t.b
Pooled	Controls (115)		–0.08 [–0.10, –0.05]	—	–0.02 [–0.10, 0.06]	—	–0.09 [–0.18, 0.01]	—	–0.08 [–0.18, 0.02]	—
	FC+IBS-C (197)		–0.32 [–0.46, –0.17]	—	–0.13 [–0.31, 0.05]	—	–0.93 [–1.19, –0.68]	—	–0.41 [–0.50, –0.26]	—
	Mean difference		–0.24 [–0.38, –0.10]	–3.25^5^	–0.11 [–0.31, 0.08]	–1.13^5^	–0.85 [–1.12, –0.57]	–6.12^5^	–0.33 [–0.52, –0.15]	–3.49^5^

1 Values are means ± SDs or means [95% CIs]. Estimation for changes in GSRS domains and totals were extracted from the reports. Pooled estimates were derived from these aggregate summaries using a random-effects model for each of the interventions and control groups. Resulting estimates were then compared using a *Z* test and 95% CI. c.t.b., compared with baseline value; c.t.t., compared with treatment value; FC, functional constipation; GSRS, Gastrointestinal Symptoms Rating Scale; IBS-C, irritable bowel syndrome with constipation.

2 Score based on 15 items using Likert scores, covering 5 domains. Domain scores can range from 1 to 7, with higher scores representing higher levels of discomfort.

3 Not included in pooled results.

4 Difference not presented in original manuscript; calculated based on published results.

5 *Z* value.

In a large international multicenter study (16), with psyllium as the positive control intervention, with a mixed case group (FC and IBS-C) and a healthy control group, the consumption of 2 green kiwifruit/d was associated with a significant reduction in GSRS abdominal pain scores in both the IBS-C group (*P* ≤ 0.024) and the FC+IBS-C group (*P* ≤ 0.027) postintervention. In the FC+IBS-C group, the difference in GSRS abdominal pain score between kiwifruit and the positive control reached significance (*P* ≥ 0.083). One trial (23) compared consumption of 2 enzymatically active gold kiwifruit with or without the skin per day in IBS-C (no healthy control group, no placebo) and reported a significant reduction in abdominal pain with the ingestion of kiwifruit flesh and kiwifruit skin with both the Birmingham IBS survey (*P* ≤ 0.004) and the GSRS (*P* ≤ 0.036) when compared with kiwifruit flesh only. An increase in the dosage of enzymatically active gold kiwifruit to 3 per day (17) (without the skin) significantly reduced abdominal pain in a combined FC+IBS-C group posttreatment (*P* ≤ 0.05), while psyllium did not (*P* > 0.05). The absence of an effect of 2 enzymatically active gold kiwifruit per day (flesh only) on abdominal pain both in comparison to psyllium and posttreatment was confirmed in a third study (18).

### Abdominal bloating and distension

Only 1 of the trials using kiwifruit supplements reported on abdominal bloating (24): there was a significant reduction in abdominal bloating after the intervention (*P* ≤ 0.003 after week 2, *P* ≤ 0.002 after week 3, and *P* ≤ 0.004 after week 4 compared with baseline). With green kiwifruit, healthy participants assessed for gas transit ingesting 2 kiwifruit/d alongside a low-flatulogenic diet showed no difference in abdominal distension compared with the group ingesting the low-flatulogenic diet alone (22). When healthy controls were supplemented acutely with green kiwifruit after ingestion of 250 g beef muscle protein, abdominal bloating was reported as being significantly less compared with the control group who ingested the enzymatically inactive gold kiwifruit variety Hort16A (49) (*P* ≤ 0.005 and *P* ≤ 0.028, using 2 different methods). A study using 3 green kiwifruit/d (21) showed a significant reduction in bloating and swelling (*P* ≤ 0.001) posttreatment. The studies with enzymatically active gold kiwifruit did not report any effect on abdominal bloating or distension.

### Disrupted swallowing and reflux

A recent, still unpublished trial from New Zealand (18) using 2 enzymatically active gold kiwifruit per day as the intervention in participants with FC and IBS-C showed significant improvement in Patient-Reported Outcome Measurement Information System (PROMIS) disrupted swallowing scores (*P* ≤ 0.036) and in SAGIS acid regurgitation and gas scores (*P* ≤ 0.001) posttreatment. However, there were no significant differences between kiwifruit and the positive control for the same scores. The SAGIS acid regurgitation and gas score includes items on belching, acid burping, and disrupted swallowing. The same study could not demonstrate a reduction in GSRS reflux scores.

However, a still unpublished international multicenter study (16) using 2 green kiwifruit/d was able to show a significant improvement in the GSRS reflux domain in the IBS-C group and the combined FC+IBS-C group alike (*P* ≤ 0.013 and *P* ≤ 0.002) posttreatment; in the FC group, the posttreatment improvement did not reach significance (*P* ≥ 0.051). The difference in GSRS reflux scores between the green kiwifruit intervention and the positive control was not significant.

### Dyspepsia

Only 3 studies reported on dyspeptic symptoms. The international multicenter study on 2 green kiwifruit/d (16) demonstrated a significant reduction in indigestion as measured by the GSRS in all participant groups posttreatment (control: *P* ≤ 0.028; FC: *P* ≤ 0.017; IBS-C: *P* ≤ 0.002; and combined FC+IBS-C: *P* ≤ 0.001). The difference between the interventions was significant in the FC group (*P* ≤ 0.043) and in the combined group (*P* ≤ 0.005) for the GSRS indigestion score.

Similarly, the trial using 3 enzymatically active gold kiwifruit/d noted a significant improvement in the GSRS indigestion score (*P* ≤ 0.002) posttreatment in the combined FC+IBS-C group, which was significantly different when compared with the positive control (*P* ≤ 0.05 posttreatment, *P* ≤ 0.025 in comparison between interventions). The study that used 2 enzymatically active gold kiwifruit/d (18) showed a significant reduction in the same score in all groups in comparison to baseline values (*P* ≤ 0.008), while the difference between the intervention and the positive control did not reach significance (*P* ≥ 0.056). In addition, SAGIS epigastric symptom scores also significantly improved (*P* ≤ 0.003) posttreatment in this study, but there was no difference between the treatments. The SAGIS epigastric symptom score includes items on postprandial and epigastric pain, fullness, early satiety, abdominal cramps, retrosternal discomfort, and bloating—all symptoms of dyspepsia (34).

### Synthesis of results

**FIGURE 2:**
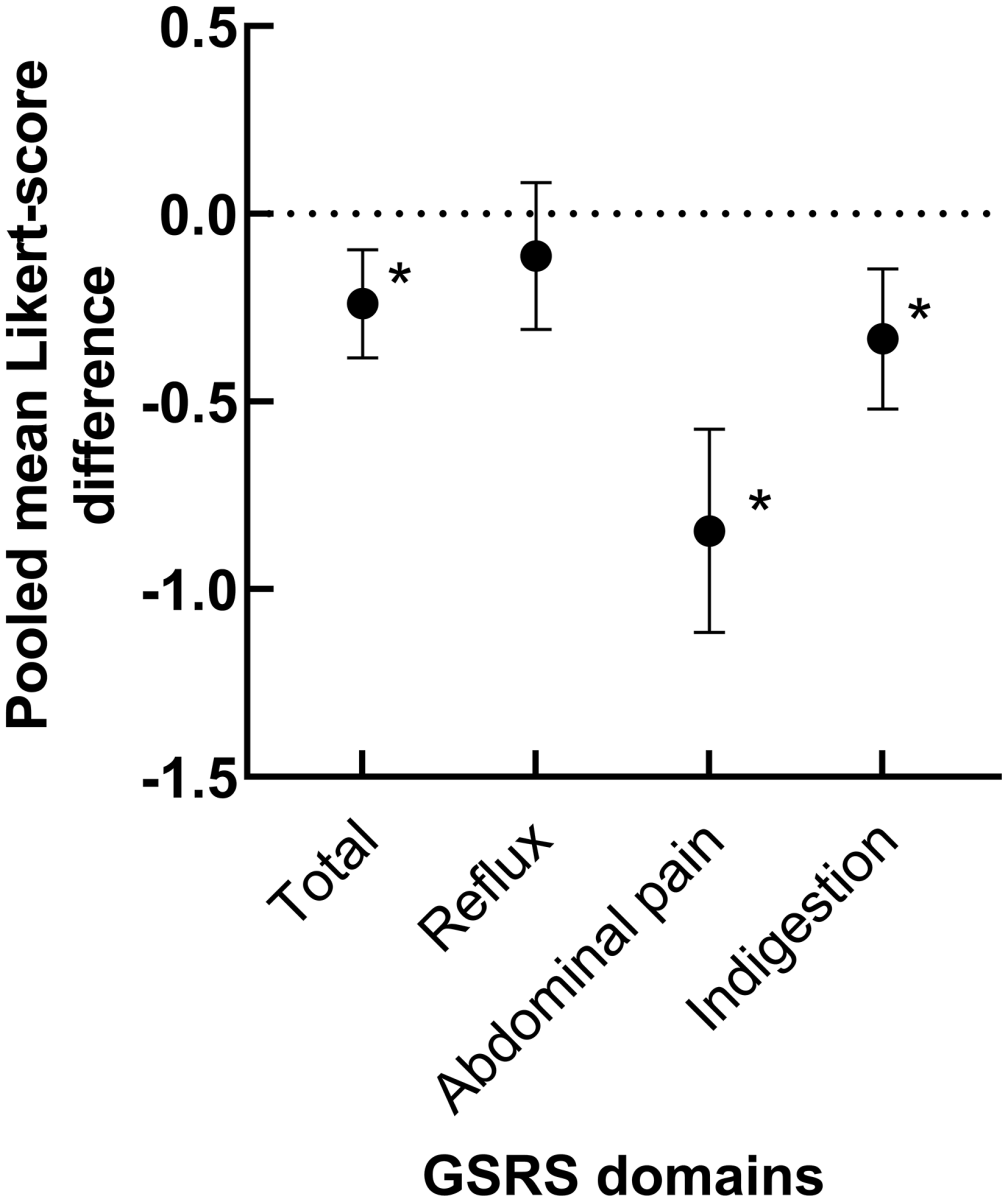
The effect of habitual kiwifruit consumption on pooled mean Likert-score differences of total GSRS reflux, abdominal pain, and indigestion domains. Scores are pooled arithmetic mean differences of cases (*n* = 197) − pooled arithmetic mean differences of controls (*n* = 115); error bars are 95% CIs (Table 3). Asterisks indicate statistical significance, *Z* value ≤ –3. Scores are based on 15 items using Likert scores. Domain scores can range from 1 to 7, with higher scores representing higher levels of discomfort. A reduction in scores indicates symptom improvement. GSRS, Gastrointestinal Symptom Rating Scale.

In summary, there is some, although sparse, evidence that both kiwifruit varieties as well as kiwifruit supplements positively affect upper GI health, as shown in **Figure 2** and **Table 4**. While the studies reported improvements in symptoms postintervention, these effects are of limited value. In the dyspepsia domain, this evidence is limited to just a few studies, but these studies are of high quality. There is some evidence that the habitual consumption of both kiwifruit varieties improves symptoms of dyspepsia. Statistically, the pooled effect on indigestion is significant (*Z* value ≤ –3.492; Figure 2, Table 3). In the case of abdominal discomfort and pain, the evidence is of good quality, especially for green kiwifruit; therefore, habitual intake of 2 green kiwifruit/d may be able to reduce abdominal discomfort or pain related to IBS-C. This is confirmed statistically, with the mean pooled difference between cases and controls reaching a *Z* value of –6.12 (Table 3). Interestingly, enzymatically active gold kiwifruit seem to be able to reduce abdominal pain and discomfort in a similar way if the skin is consumed as well, or the daily dose is increased to 3 gold kiwifruit. For abdominal distension and bloating, the evidence is limited to 2 of the studies conducted acutely (1 d) in healthy participants (*n* = 20), or for the habitual intake of 3 kiwifruit for 3 wk, in a good-quality study. As a result, that green kiwifruit are able to promote digestion and reduce abdominal distension and bloating when ingested after a meal may be a valid claim, but the prolonged improvement in abdominal bloating and distension through habitual intake over longer periods cannot be completely ascertained yet due to a lack of evidence. There is no evidence that enzymatically active gold kiwifruit have a similar effect, neither with acute nor habitual consumption.

**TABLE 4:** Summary of evidence found in support of kiwifruit and kiwifruit supplements benefitting upper gastrointestinal symptoms^1^

	Evidence in support of an effect on			
	Abdominal discomfort and pain	Abdominal distension and bloating	Disrupted swallowing and reflux	Indigestion/dyspepsia
Supplements				
Studies, *n*	3	1	0	0
Quality	Low	Medium	N/A	N/A
Evidence	Uncertain	Medium	None	None
Green kiwifruit				
Studies, *n*	3	3	1	1
Quality	Medium–high	Medium	High	High
Evidence	Good	Medium	Good	Good
Gold kiwifruit				
Studies, *n*	2	0	1	2
Quality	Medium	N/A	Medium	High
Evidence	Low	None	Low	Good
Summary	Good evidence for green, seemingly dose dependent for gold	Medium evidence for green in acute setting, medium for habitual intake, and absent for gold	Evidence is limited for both varieties, needs to be expanded	Evidence is of high quality but limited number of studies, needs to be expanded

1 N/A, not available.

## Discussion

### Summary of evidence

In this scoping review, we identified 12 clinical trials that addressed symptom relief of upper GI symptoms through kiwifruit or kiwifruit supplement intake, published (and unpublished) between 1946 and January 2021. The results provide evidence for upper GI symptom relief by kiwifruit intake, but also a lack of research focused on kiwifruit and upper GI symptom relief, a paucity of common trial protocols, as well as limited use of standardized, validated patient-reported outcome tools. We found that, across the 5 trials using standardized outcome tools, there is robust evidence that kiwifruit intake has a positive influence on abdominal pain and dyspepsia, which may be either dose dependent or dependent on the type of kiwifruit that is ingested. Overall, included studies support the notion that kiwifruit intake has positive effects on upper GI symptoms, but details remain unclear.

### Limitations

This scoping review has some limitations. The research strategy was developed by the primary author and not peer-reviewed. In addition, the primary author was the sole reviewer of the identified reports, which may have introduced bias to this review and affected the data-charting process and the critical evaluation of the quality of the identified reports.

Another severe limitation of this review is the scarcity of standardized protocols in many studies, common in studies on GI health (4). The trial phases vary in length, participant groups are often not well defined, or use different methods to characterize their cohorts. The participant numbers are often small, and the clinical measurement tools are often not validated, or not comparable, or are altered or designed by the researchers themselves. This makes it difficult to compare and to evaluate the quality of these reports. Further, the upper GI symptoms were unexpected outcomes, and therefore standardization was not possible as such. In addition, the number of included studies using a similar outcome measurement tool was very limited, which made it necessary to pool the data. This will have likely affected the statistical validity of the reported GRSR domain outcomes.

Another difficulty for comparison of studies is the variety of placebos used, which included positive controls such as prunes and psyllium (16–18, 25), negative controls such as glucose (59) or maltodextrin (60), the absence of a control (22), and comparable products based on the gold kiwifruit variety Hort16 (47, 49, 61, 62). In these cases, the hypotheses of the studies need to be taken into account, such as digestion of protein by actinidin, or alterations of gut flora by increased intake of easily fermentable sugars and fibers. However, since the main research interest of this study lies in the influence of habitual kiwifruit consumption on upper GI symptoms, and since two-thirds of the included studies had a crossover design where the effects of kiwifruit consumption were primarily compared with baseline, the possible effects of placebos are negligible.

We also acknowledge that this review was focused primarily on upper GI symptoms reported as secondary outcomes in studies focused on constipation symptom relief. The majority of the participants experienced upper GI symptoms in addition to lower GI symptoms; this will negatively influence the generalization of the results, since the participants had more than 1 symptom.

### Conclusions

Based on the review of 12 studies, we found that there is consistent evidence that kiwifruit ingestion improves upper GI symptoms. Specifically, the habitual ingestion of green or gold kiwifruit may reduce abdominal pain and, to a lesser degree, symptoms of dyspepsia.

To further define the effects of kiwifruit ingestion on upper GI health and symptom relief, more research is needed. Clinical trials need to be standardized, with well-defined cohorts, medium to large populations, and using validated surveys focused on upper GI symptom relief. The inclusion of a cohort with symptoms of dyspepsia would be recommended, or a large systems-biology study observing all aspects of the microbiota-gut-brain axis after kiwifruit intake.

### Funding

Of the identified studies, 91.7% (*n* = 11) were funded or partially supported through the industry, 50% (*n* = 5) received additional public funding, 8.3% (*n* = 1) did not disclose funding, and 8.3% (*n* = 1) relied solely on public funding. This systematic scoping review was funded by Zespri International Ltd. Zespri personnel were not involved in the conduct, analysis, or reporting of the scoping review.

## Data Availability

Data described in the manuscript will be made available upon request to the corresponding author.

## Notes

Supported by Zespri International Ltd.

Author disclosures: RBG has served on advisory boards of Zespri International Ltd., and all authors have received funding for the completion of the commissioned review. Zespri personnel participated in discussions on review design with the authors, but were not involved in the conduct, analysis, or reporting of the scoping review.

Abbreviations used: FC, functional constipation, FGID, functional gastrointestinal disorder; GI, gastrointestinal; GSRS, Gastrointestinal Symptom Rating Scale; IBS, irritable bowel syndrome; IBS-C, irritable bowel syndrome with constipation; SAGIS, Structured Assessment of Gastrointestinal Symptoms.
